# Computational Identification of Tumor Suppressor Genes Based on Gene Expression Profiles in Normal and Cancerous Gastrointestinal Tissues

**DOI:** 10.1155/2020/2503790

**Published:** 2020-07-22

**Authors:** Qingrong Sun, Md. Nazim Uddin, Mengyuan Li, Xiaosheng Wang, Maode Lai

**Affiliations:** ^1^Department of Basic Medicine, School of Basic Medicine and Clinical Pharmacy, China Pharmaceutical University, Nanjing 211198, China; ^2^Cancer Genomics Research Center, School of Basic Medicine and Clinical Pharmacy, China Pharmaceutical University, Nanjing 211198, China; ^3^Biomedical Informatics Research Lab, School of Basic Medicine and Clinical Pharmacy, China Pharmaceutical University, Nanjing 211198, China

## Abstract

Cancer prevails in various gastrointestinal (GI) organs, such as esophagus, stomach, and colon. However, the small intestine has an extremely low cancer risk. It is interesting to investigate the molecular cues that could explain the significant difference in cancer incidence rates among different GI tissues. Using several large-scale normal and cancer tissue genomics datasets, we compared the gene expression profiling between small intestine and other GI tissues and between GI cancers and normal tissues. We identified 17 tumor suppressor genes (TSGs) which showed significantly higher expression levels in small intestine than in other GI tissues and significantly lower expression levels in GI cancers than in normal tissues. These TSGs were mainly involved in metabolism, immune, and cell growth signaling-associated pathways. Many TSGs had a positive expression correlation with survival prognosis in various cancers, confirming their tumor suppressive function. We demonstrated that the downregulation of many TSGs was associated with their hypermethylation in cancer. Moreover, we showed that the expression of many TSGs inversely correlated with tumor purity and positively correlated with antitumor immune response in various cancers, suggesting that these TSGs may exert their tumor suppressive function by promoting antitumor immunity. Furthermore, we identified a transcriptional regulatory network of the TSGs and their master transcriptional regulators (MTRs). Many of MTRs have been recognized as tumor suppressors, such as HNF4A, ZBTB7A, p53, and RUNX3. The TSGs could provide new molecular cues associated with tumorigenesis and tumor development and have potential clinical implications for cancer diagnosis, prognosis, and treatment.

## 1. Introduction

The gastrointestinal (GI) tract is a highly organized organ system in human with many important functions, such as the absorption of food and nutrients, endocrine secretion, and resistance to microorganism invasion. The GI cancers prevail and account for a large number of cancer deaths worldwide, of which colorectal, stomach, esophagus, liver, and pancreatic cancers are the most common [1]. However, the small intestine, an important GI tract organ for the food and nutrients absorption, has much lower cancer incidence compared to other GI tract organs. Therefore, it is interesting to investigate what molecular features may explain the significantly lower cancer incidence rate in small intestine than in other GI tract organs. With the advancement of genomics technology, a large volume of cancer and normal tissue genomics data has been produced that would enable us to investigate the association between genomic features and the significantly differential cancer incidence rates among different human tissues.

**Figure 1:**
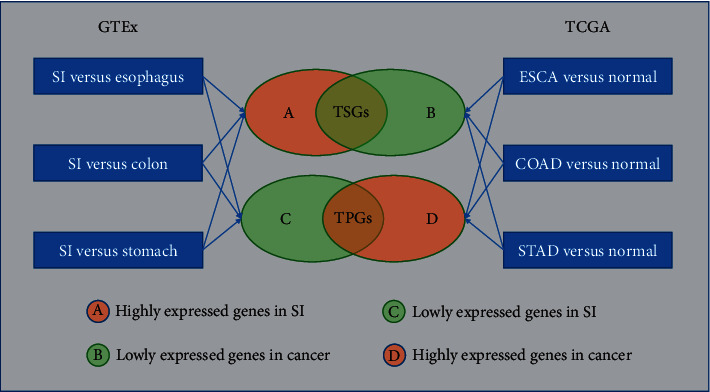
The analysis pipeline for identifying tumor promoter genes (TPGs) and tumor suppressor genes (TSGs). The differentially expressed genes (DEGs) between two classes of samples were identified using the threshold of Student's *t*-test adjusted *P* value (FDR) <0.05 and mean gene-expression fold-change >1.5. The FDR was calculated by the Benjamini and Hochberg (BH) method [6]. SI: small intestine; FDR: false discovery rate.

In this study, we compared the gene expression profiling between the tissue (small intestine) with a low cancer risk and the tissues (colon, stomach, and esophagus) with a high cancer risk using the Genotype-Tissue Expression (GTEx) data [2]. We identified the genes that were significantly differentially expressed between both groups of tissues. Moreover, we compared the gene expression profiling between cancer and normal tissues in colon, stomach, and esophagus cancer cohorts using The Cancer Genome Atlas (TCGA) data and identified differentially expressed genes (DEGs) between cancer and normal tissues. We obtained common DEGs between the results from both analyses and divided them into tumor promoter genes (TPGs) and tumor suppressor genes (TSGs). The TPGs were those that are more highly expressed in colon, stomach, and esophagus than in small intestine and more highly expressed in colon, stomach, and esophagus cancers than in their normal tissues. By contrast, the TSGs were those that are more lowly expressed in colon, stomach, and esophagus than in small intestine and more lowly expressed in colon, stomach, and esophagus cancers than in their normal tissues.  Figure 1 is a summary of the analysis pipeline for identifying TPGs and TSGs. Furthermore, we verified these results using several Gene Expression Omnibus (GEO) datasets [3]. The downstream analysis of the genes identified was performed based on TCGA data.

## 2. Materials and Methods

### 2.1. Materials

The gene expression profiling of normal tissues (small intestine, colon, stomach, and esophagus) was downloaded from GTEx (https://gtexportal.org/home/) and GEO (https://www.ncbi.nlm.nih.gov/gds) databases. The gene expression profiling of colon, stomach, and esophagus cancers and their normal tissues were downloaded from TCGA (https://portal.gdc.cancer.gov/). In addition, we obtained the gene expression profiling and clinical immunotherapy response data of two melanoma cohorts (Nathanson et al. [4] and Roh et al. [5]) from the associated publications. The sample size of cancer and normal tissues are presented in Supplementary Table S1.

### 2.2. Identification of Differentially Expressed Genes

We identified differentially expressed genes (DEGs) between two classes of samples using Student's *t*-test. The false discovery rate (FDR) estimated by the Benjamini and Hochberg (BH) method [6] was used to adjust for multiple tests. The threshold of FDR <0.05 and mean gene-expression fold-change >1.5 was used to identify the DEGs between two classes of samples.

### 2.3. Identification of TPGs and TSGs

Based on the GTEx datasets, we identified three sets of more highly expressed genes and three sets of more lowly expressed genes in small intestine by comparing small intestine tissue versus colon tissue, intestine tissue versus gastric tissue, and intestine tissue versus esophagus tissue. We obtained the set of genes common in the three sets of more highly expressed genes in small intestine (termed as SI-HEGs) and the set of genes common in the three sets of more lowly expressed genes in small intestine (termed as SI-LEGs). Furthermore, based on the TCGA datasets, we identified three sets of upregulated genes and three sets of downregulated genes in cancer by comparing colon cancer versus colon tissue, gastric cancer versus gastric tissue, and esophagus cancer versus esophagus tissue. We termed the set of genes consistently upregulated in the three cancer types as Ca-HEGs and the set of genes consistently downregulated in the three cancer types as Ca-LEGs. We defined TPGs as the set of genes overlapping between SI-LEGs and Ca-HEGs and TSGs as the set of genes overlapping between SI-HEGs and Ca-LEGs.

### 2.4. Survival Analyses

We compared overall survival (OS) and disease-free survival (DFS) between cancer patients with higher gene expression levels (>median) and cancer patients with lower gene expression levels (<median) and between cancer patients with higher tumor immunity (>median) and cancer patients with lower tumor immunity (<median). The tumor immunity was calculated by the ABSOLUTE algorithm [7]. The significance of survival time differences was evaluated by the log-rank test using a threshold of *P* < 0.05. Kaplan–Meier curves were used to show the survival time differences. The survival analyses were performed in the TCGA datasets.

### 2.5. Correlation of Gene Expression Levels with Tumor Purity, Tumor Immune Cell Infiltration Levels, and Immunotherapy Response

We evaluated the correlations of gene expression levels [8] with tumor purity and the abundance of tumor immune cell (CD8+ T cells and dendritic cells) infiltration using TIMER [9]. In both melanoma cohorts (Nathanson et al. [4] and Roh et al. [5]), we divided cancer samples into two groups based on the median expression levels of TSGs and compared the immunotherapy response rates between them.

### 2.6. Comparison of the Methylation Levels of TSGs between Tumor and Normal Tissues

We compared the mean gene promoter methylation levels (*β* values) between tumor and normal tissues in 18 TCGA cancer types and used the linear regression model to evaluate the correlation between gene expression levels and mean gene promoter methylation levels in these cancer types using MethHC [10].

### 2.7. Identification of Master Transcriptional Regulators (MTRs) of TSGs

We used iRegulon [11], a Java-based plugin in Cytoscape, to identify the MTRs that regulate the TSGs. iRegulon uses a large collection of transcription factor (TF) motifs and ChIP-seq tracks to identify the target genes of TFs on the basis of the normalized enrichment score (NES).

## 3. Results

### 3.1. Identification of TSGs

We identified a total of 17 TSGs and zero TPGs. The 17 TSGs included *FAM177B*, *TMEM25*, *ANGPTL6*, *TREH*, *RASGRP2*, *SLC28A2*, *MADCAM1*, *XPNPEP2*, *SEPP1*, *CCL21*, *PNOC*, *GUCA2B*, *GSTA2*, *G6PC*, *FCER2*, *CBFA2T3*, and *BMP5*. Among the 17 TSGs, 8 genes were found to be associated with certain KEGG [12] pathways (Table 1). These pathways are mainly involved in metabolism (starch and sucrose, cytochrome P450, glutathione, galactose, and glycolysis), immune (chemokine signaling, cytokine-cytokine receptor interaction, intestinal immune network for IgA production, adipocytokine, insulin signaling, and hematopoietic cell lineage), and cell proliferation, differentiation, and migration (MAPK signaling, cell adhesion molecules, and TGF-*β* signaling). These results indicate that these gene signatures are importantly involved in tumor suppression by the regulation of metabolism, immune, and cell growth signaling-associated pathways.

**Figure 2:**
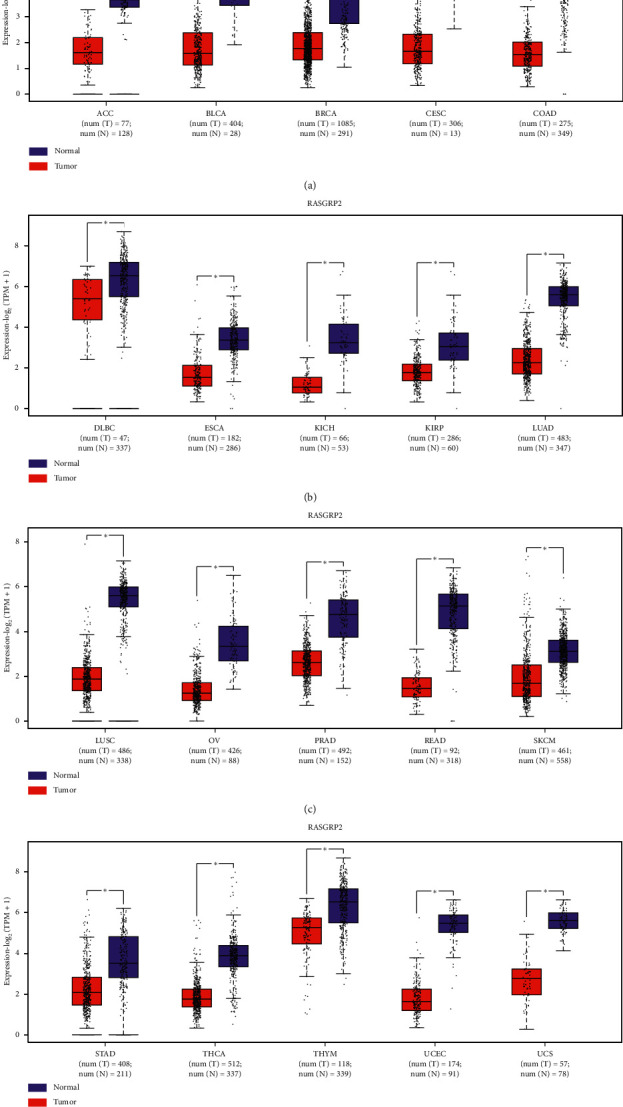
*RASGRP2* is downregulated in cancer. *RASGRP2* has significantly lower expression levels in various cancers than in normal tissues (Student's *t*-test, *P* < 0.001). TPM: transcripts per kilobase million. ^*∗∗∗*^*P* < 0.001; it also applies to the following figures.

Moreover, we found that a majority of the 17 TSGs showed significantly lower expression levels in various cancers apart from colon, stomach, and esophagus cancers than in normal tissues. For example, *RASGRP2* was downregulated in 20 TCGA cancer types (Figure 2). *CCL21* had lower expression levels in 18 TCGA cancer types (Supplementary Figure S1). *CBFA2T3* and *XPNPEP2* were downregulated in 15 and 10 TCGA cancer types (Supplementary Figures S2 and S3). These results suggest that the TSGs may play crucial roles in tumor suppression in a wide variety of cancer types.

**Figure 3:**
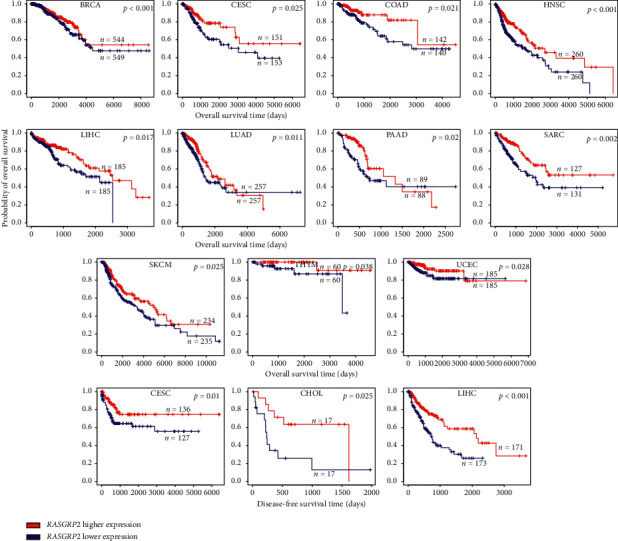
Downregulation of *RASGRP2* is associated with a worse survival prognosis in various cancers. Kaplan–Meier survival curves show that the lower expression of *RASGRP2* is associated with a worse overall or disease-free survival in various cancers (log-rank test, *P* < 0.05).

Furthermore, we found that the elevated expression of many TSGs correlated with a better survival prognosis in various cancers. For example, the upregulation of *RASGRP2* was consistently associated with a better overall survival (OS) or disease-free survival (DFS) in 12 cancer types, including breast invasive carcinoma (BRCA), cervical and endocervical cancers (CESC), cholangiocarcinoma (CHOL), colon adenocarcinoma (COAD), head and neck squamous cell carcinoma (HNSC), liver hepatocellular carcinoma (LIHC), lung adenocarcinoma (LUAD), pancreatic adenocarcinoma (PAAD), sarcoma (SARC), skin cutaneous melanoma (SKCM), thymoma (THYM), and uterine corpus endometrial (UCEC) (Figure 3). The elevated expression of *CBFA2T3* was consistently associated with a better OS or DFS in 10 cancer types, including CHOL, COAD, HNSC, kidney chromophobe (KICH), brain lower-grade glioma (LGG), LIHC, LUAD, SARC, thyroid carcinoma (THCA), and THYM (Supplementary Figure S4). Again, these results suggest the tumor suppressive function of TSGs.

The depressed expression of TSGs is associated with the hypermethylation of these genes in cancer.

We compared the DNA methylation levels of TSGs between cancer and normal tissues and found that many TSGs exhibited significantly higher methylation levels in various cancers. For example, the methylation levels of *RASGRP2* promoter were significantly higher in 18 TCGA cancer types than in their normal tissues (Figure S5). The promoter region of *CCL21* had significantly higher methylation levels in 17 TCGA cancer types (Supplementary Figure S7). Furthermore, linear regression analysis showed that the methylation levels of TSGs had a significant inverse correlation with the expression levels of TSGs in many cancer types. For example, *RASGRP2* methylation levels significantly inversely correlated with its expression levels in stomach adenocarcinoma (STAD), PAAD, and prostate adenocarcinoma (PRAD) with the absolute correlation coefficient not less than 0.3 (Supplementary Figures S6 and S7). In addition, previous studies have shown that several TSGs had significantly higher methylation levels in cancer, such as *CBFA2T3* [13] and *TMEM25* [14]. These results suggest that the downregulation of many TSGs is associated with their elevated methylation levels in cancer.

The higher expression levels of TSGs are associated with lower tumor purity and more active antitumor immune response in cancer.

**Figure 4:**
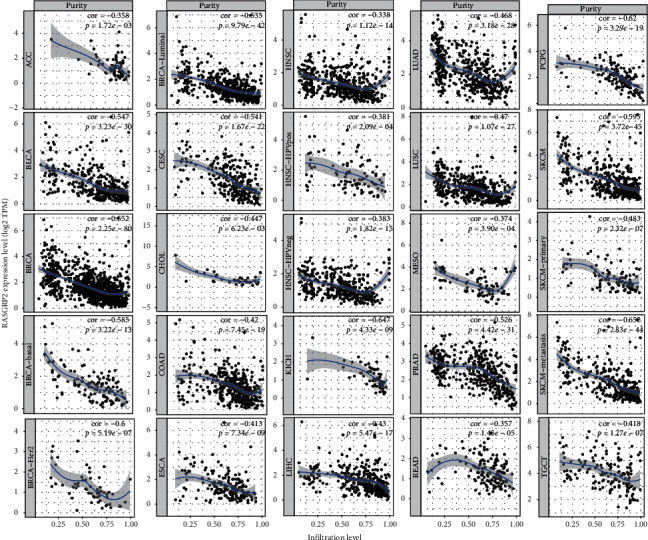
Correlations of the expression levels of tumor suppressor genes (TSGs) with tumor purity in cancer. The *RASGRP2* expression levels were inversely associated with tumor purity in 20 TCGA cancer types/subtypes.

**Figure 5:**
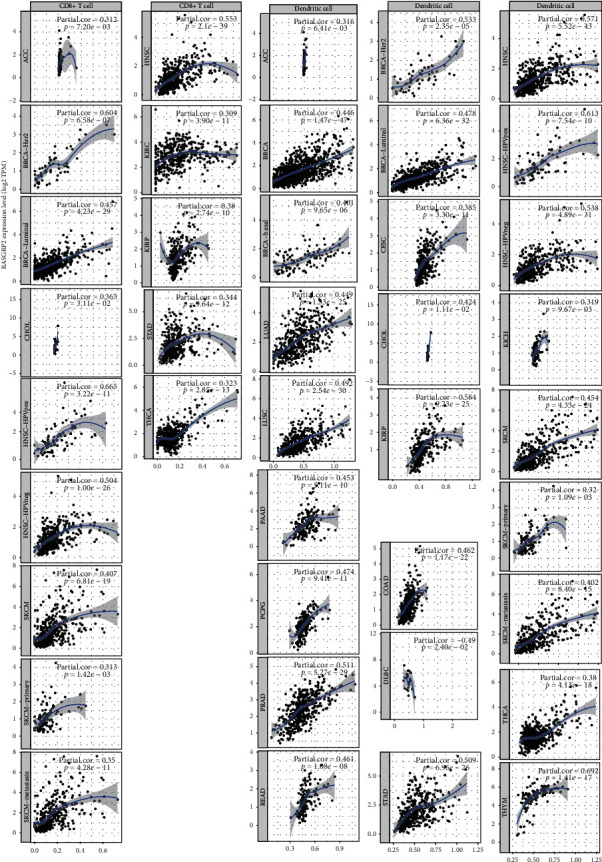
Correlations of the expression levels of tumor suppressor genes (TSGs) with immune cell infiltration levels in cancer. The *RASGRP2* expression levels were positively associated with the enrichment levels of immune cells (CD8+ T cells and dendritic cells) in multiple TCGA cancer types/subtypes. Spearman's rank correlation test *P* values and correlation coefficients are shown. RSEM : RNA-Seq by Expectation Maximization [8].

We found that the expression levels of TSGs tended to inversely correlate with tumor purity in various cancers. For example, the *RASGRP2* expression levels were inversely associated with tumor purity in 25 TCGA cancer types/subtypes with the Spearman rank correlation coefficient (*cor*) not greater than −0.3 (Figure 4). The expression levels of *CCL21* had a significant inverse correlation with tumor purity in 17 TCGA cancer types (*cor* ≤−0.3) (Supplementary Figure S8). These results indicate that the higher expression levels of TSGs may correlate with more nontumor components in cancer. In fact, the expression levels of TSGs were likely to have a positive correlation with antitumor immune signatures in various cancers. For example, the *RASGRP2* expression levels positively correlated with the enrichment levels of CD8+ T cells in 14 TCGA cancer types/subtypes and with the enrichment levels of dendritic cells in 26 TCGA cancer types/subtypes (*cor* ≥0.3) (Figure 5). The expression levels of *CCL21* had a significant positive correlation with the enrichment levels of CD8+ T cells and dendritic cells in 5 and 6 TCGA cancer types, respectively (*cor* ≥0.3) (Supplementary Figure S9). Previous studies also showed that the expression of certain TSGs could promote antitumor immunity in diverse cancers, such as *CCL21* [15–17], *MADCAM1* [18], and *FCER2* [19]. Again, these results suggest that the elevated expression of TSGs is associated with a favorable prognosis in cancer since the higher levels of tumor-infiltrating lymphocytes (TILs) are associated with improved survival in cancer patients [20, 21].

Since the elevated expression of TSGs was associated with the higher levels of TILs in tumor and the TILs levels were a positive predictor for cancer immunotherapy response (ITR) [22], the expression levels of TSGs could be positively associated with ITR in cancer. To prove this hypothesis, we examined the correlation between the expression levels of TSGs and ITR in two cancer (melanoma) cohorts (Nathanson et al.'s cohort [4] and Roh et al.'s cohort [5]) with anti-CTLA-4/PD-1 immunotherapy. We found that the higher *GSTA2* expression levels were associated with a significantly higher ITR in Nathanson et al.'s cohort (Fisher's exact test, *P*=0.036, OR = 8.91) and that the expression of *CCL21* and *MADCAM1* positively correlated with ITR in Roh et al.'s cohort (Fisher's exact test, *P*=0.002, OR = 8.07 for *CCL21* and *P*=0.058, OR = 4.55 for *MADCAM1*) (Table 2). These results proved the hypothesis that the expression of TSGs is capable of promoting ITR in cancer.

### 3.2. Master Transcriptional Regulators (MTRs) of TSGs

**Figure 6:**
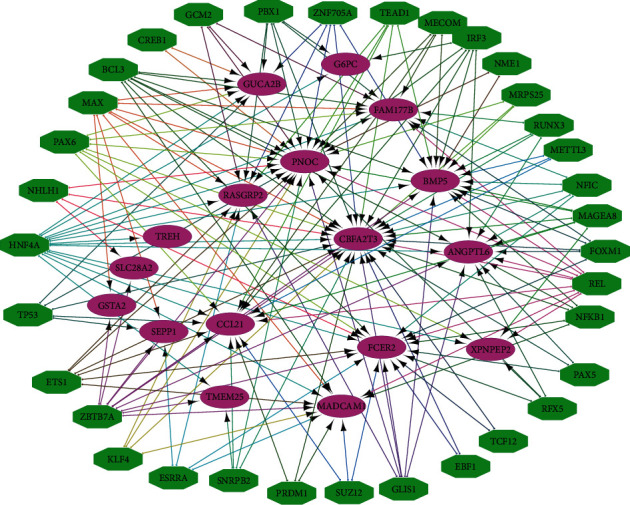
Regulatory network of 17 tumor suppressor genes (TSGs) and their master transcriptional regulators (MTRs). The purple nodes indicate TSGs and the green nodes indicate MTRs.

To understand the regulatory mechanism underlying the different cancer risk in varying tissues, we identified 34 MTRs that significantly regulated the 17 TSGs (NES >3) (Figure 6). Among the 34 MTRs, HNF4A (hepatocyte nuclear factor 4 alpha) was the most highly enriched which regulated 14 TSGs. HNF4A plays a role in the development of multiple organs including intestines, liver, and kidney and functions as a repressor of cell proliferation [23]. The reduced expression of HNF4A has been associated with tumorigenesis in various cancers [24, 25]. This is consistent with the tumor suppressive function of the TSGs regulated by HNF4A. The second highly enriched MTR of TSGs was ZBTB7A regulating 11 TSGs. ZBTB7A acts as a tumor suppressor by repressing the expression of key genes in tumor glycolysis [26] and negatively regulating TGF-*β* pathway [27]. Previous studies have revealed its tumor suppressor role in a wide variety of cancers [28–30]. Again, this is in line with the tumor suppressive function of the TSGs regulated by ZBTB7A. Some other MTRs have been also identified as tumor suppressors, such as p53 [31–33] and RUNX3 [34–36]. Collectively, these results suggest that the identification of MTRs of TSGs may provide insights into the regulatory mechanism underlying the different cancer risks in different tissues and cancer development.

## 4. Discussion

Cancer prevails in variously different human organs, such as lung, colon, stomach, esophagus, liver, pancreas, brain, head and neck, breast, and kidney. However, some human organs have an extremely low cancer risk, such as small intestine, spleen, and heart. Although a recent study has proposed that the total number of stem cell divisions largely explained the cancer risk in different tissues [37], it cannot explain why some tissues with a large number of stem cell divisions have a low cancer risk, such as small intestine. Thus, it is necessary to investigate the other molecular cues that could explain the different cancer risk among different tissues. We identified 17 TSGs which showed significantly higher expression levels in small intestine than in other GI tissues including esophagus, stomach, and colon. Moreover, these genes were more lowly expressed in GI cancers than in GI normal tissues and were also downregulated in many other cancer types relative to their normal control, suggesting their tumor suppressor role. The tumor suppressive function of these TSGs was further confirmed by the fact that the elevated expression of many TSGs was associated with a better survival prognosis in various cancers. Furthermore, we revealed that the downregulation of many TSGs was associated with their promoter hypermethylation in cancer, demonstrating the important relationship between DNA methylation and cancer [38]. Pathway analysis showed that these TSGs were mainly involved in metabolism, immune, and cell growth signaling-associated pathways. Interestingly, the expression of many TSGs inversely correlated with tumor purity and positively correlated with antitumor immune cell infiltration levels in a wide variety of cancers, suggesting that these TSGs may exert their tumor suppressive function by promoting antitumor immunity. Moreover, the higher expression levels of certain TSGs, including *GSTA2*, *CCL21*, and *MADCAM1*, were associated with a significantly higher ITR in cancer. This could be attributed to the higher levels of TILs in the tumors highly expressing these TSGs. In addition, we identified 34 MTRs of TSGs, many of which have been recognized as tumor suppressors, such as HNF4A, ZBTB7A, p53, and RUNX3. It suggests that the transcriptional regulatory network could also be an important approach whereby the TSGs inhibit tumor development.

Our results showed that the elevated expression of TSGs was likely associated with better survival prognosis in cancer. A possible explanation is that the expression of TSGs is positively associated with the infiltration of TILs which may improve the immune response against cancer cells. Our results also showed that the elevated expression of TSGs was likely associated with lower tumor purity whose association with prognosis is controversial. We analyzed the correlation between tumor purity and survival prognosis in 33 TCGA cancer types. We found that tumor purity had a significant positive correlation with OS in COAD, KIRP, and PRAD and with DFS in kidney renal clear cell carcinoma (KIRC), UCEC, and uveal melanoma (UVM) (log-rank test, *P* < 0.05), but have a significant negative correlation with DFS in HNSC (*P*=0.035) (Supplementary Figure S10). These results indicate that the correlation between tumor purity and survival prognosis was not significant in most cancer types although it was positive or negative in a few cancer types.

**Figure 7:**
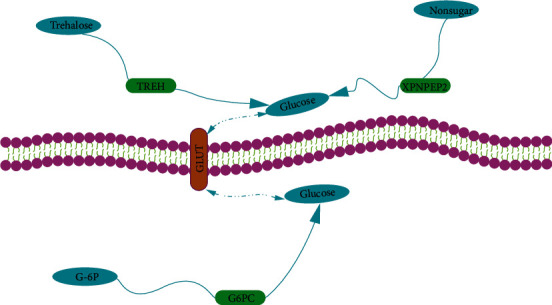
Three tumor suppressors (*G6PC*, *XPNPEP2*, and *TREH*) are involved in glucose metabolism in cells.

Based on another gene expression profiling dataset from GEO [3], we found that three TSGs (*G6PC*, *XPNPEP2*, and *TREH*) had significantly higher expression levels in small intestine tissue than in colon and stomach tissues, confirming the results obtained from the analysis of GTEx dataset. Using COMPARTMENTS [39], we found that G6PC was located in the endoplasmic reticulum and both XPNPEP2 and TREH were located in the plasma membrane. On the basis of the connections between pathways and proteins' subcellular location, we found that both G6PC and TREH were associated with glucose metabolism and contributed to the elevation of glucose levels. In addition, XPNPEP2 acts as a proline-specific aminopeptidase to regulate the proline concentration which is capable of affecting the glucose concentration [40]. Thus, all the three proteins can enhance the glucose concentration both inside and outside cells to inhibit glucose metabolism in cells (Figure 7). Again, these results suggest that the metabolic regulation may play a crucial role in controlling tumorigenesis and tumor development.

A literature review showed that many of the 17 TSGs have been associated with tumor suppression. For example, *BMP5* (bone morphogenetic protein 5) was downregulated in breast tumors relative to normal tissues and its downregulation was associated with cancer recurrence [41]. This gene plays a role in tumor suppression via repressing TGF-*β*1-induced epithelial-to-mesenchymal transition [41]. *CCL21* (C-C motif chemokine ligand 21) is a cytokine gene involved in immunoregulation and inflammation. *CCL21* is able to exert antitumor immunity by activating both innate and adaptive immune responses [42, 43]. The other TSGs, such as *SEPP1* [44], *TMEM25* [45], *XPNPEP2* [46], and *G6PC* [47], have been reported to play a role in tumor suppression. These prior studies lend support to our results that these TSGs are likely to be tumor suppressor genes, although further experimental verification is needed.

In conclusion, this study provides new molecular cues associated with tumorigenesis and tumor development. The identified TSGs have potential clinical implications for cancer diagnosis, prognosis, and treatment.

## Figures and Tables

**Table 1:** The 17 tumor suppressor genes (TSGs) and their associated pathways.

Entrez ID	Symbol	Name	Pathway
11181	*TREH*	Trehalase (brush-border membrane glycoprotein)	Starch and sucrose metabolism
10235	*RASGRP2*	RAS guanyl releasing protein 2 (calcium and DAG-regulated)	Chemokine signaling pathway
			MAPK signaling pathway
8174	*MADCAM1*	Mucosal vascular addressin cell adhesion molecule 1	Cell adhesion molecules (CAMs)
			Intestinal immune network for IgA production
6366	*CCL21*	Chemokine (C-C motif) ligand 21	Chemokine signaling pathway
			Cytokine-cytokine receptor interaction
2939	*GSTA2*	Glutathione S-transferase alpha 2	Drug metabolism-cytochrome P450
			Glutathione metabolism
			Metabolism of xenobiotics by cytochrome P450
2538	*G6PC*	Glucose-6-phosphatase, catalytic subunit	Adipocytokine signaling pathway
			Galactose metabolism
			Glycolysis/gluconeogenesis
			Insulin signaling pathway
			Starch and sucrose metabolism
2208	*FCER2*	Fc fragment of IgE, low affinity II, receptor for CD23	Hematopoietic cell lineage
653	*BMP5*	Bone morphogenetic protein 5	TGF-beta signaling pathway

**Table 2:** Correlations of the expression levels of tumor suppressor genes (TSGs) with immunotherapy response in cancer. Anti-CTLA-4/PD-1 immunotherapy response rates in melanoma patients with high or low expression of three TSGs. Fisher's exact test *P* values are shown.

Cohorts	Nathanson et al.'s cohort		Roh et al.'s cohort		Roh et al.'s cohort	
Gene symbol	*GSTA2*		*MADCAM1*		*CCL21*	
Expression	Low	High	Low	High	Low	High
Response (%)	16.67	66.67	29.73	66.67	16.00	61.90
No response (%)	83.33	33.33	70.27	33.33	84.00	38.10
Fisher's exact test *P* values	0.036		0.058		0.002	

## Data Availability

The gene expression profiling of normal tissues (small intestine, colon, stomach, and esophagus) was downloaded from GTEx (https://gtexportal.org/home/) and GEO (https://www.ncbi.nlm.nih.gov/gds) databases. The gene expression profiling of colon, stomach, and esophagus cancers and their normal tissues was downloaded from TCGA (https://portal.gdc.cancer.gov/). In addition, we obtained the gene expression profiling and clinical immunotherapy response data of two melanoma cohorts (Nathanson et al.'s cohort [4] and Roh et al.'s cohort [5]) from the associated publications.

## Abbreviations

BH: Benjamini and Hochberg
BRCA: Breast invasive carcinoma
CHOL: Cholangiocarcinoma
COAD: Colon adenocarcinoma
DEGs: Differentially expressed genes
DFS: Disease-free survival
DLBC: Lymphoid neoplasm diffuse large B-cell lymphoma
ESCA: Esophageal carcinoma
FDR: False discovery rate
GEO: Gene Expression Omnibus
GI: Gastrointestinal
GTEx: The Genotype-Tissue Expression
HNSC: Head and neck squamous cell carcinoma
ITR: Immunotherapy response
KIRC: Kidney renal clear cell carcinoma
KIRP: Kidney renal papillary cell carcinoma
LGG: Brain lower-grade glioma
LIHC: Liver hepatocellular carcinoma
LUAD: Lung adenocarcinoma
MTRs: Metabolic transcriptional regulators
NES: Normalized enrichment score
OS: Overall survival
PAAD: Pancreatic adenocarcinoma
SARC: Sarcoma
SI-HEGs: Highly expressed genes in small intestine
SI-LEGs: Lowly expressed genes in small intestine
SKCM: Skin cutaneous melanoma
STAD: Stomach adenocarcinoma
TCGA: The Cancer Genome Atlas
TF: Transcription factor
THCA: Thyroid carcinoma
THYM: Thymoma
TILS: Tumor-infiltrating lymphocytes
TPGs: Tumor promoter genes
TSGs: Tumor suppressor genes
UCEC: Uterine corpus endometrial carcinoma.

## Supplementary Materials

Supplementary Materials
*Table S1*. Sample size of cancer and normal tissues in the datasets used in this study. *Figure S1*. *CCL21* tends to be downregulated in cancer. *Figure S2*. *CBFA2T3* tends to be downregulated in cancer. *Figure S3*. *XPNPEP2* tends to be downregulated in cancer. *Figure S4*. Downregulation of tumor suppressor genes (TSGs) is associated with a worse survival prognosis in various cancers. *Figure S5*. The methylation levels of *RASGRP2* promoter are higher in various cancer types than in normal tissues. *Figure S6*. The methylation levels of *RASGRP2* promoter are inversely associated with the expression levels of *RASGRP2* in cancer. *Figure S7*. The *CCL21* promoter methylation levels are significantly upregulated in 16 TCGA cancer types compared to their normal tissues. *Figure S8*. Correlations of the expression levels of *CCL21* with tumor purity in cancers. *Figure S9*. Correlations of the expression levels of CCL21 with immune cell infiltration levels in cancers. *Figure S10*. Correlation of tumor purity with survival prognosis in cancer.
