# Supplementary figures and images for: Computational Identification of Tumor Suppressor Genes Based on Gene Expression Profiles in Normal and Cancerous Gastrointestinal Tissues
Source: J Oncol. 2020 Jul 22;2020:2503790. doi: 10.1155/2020/2503790 (PMC7396062; doi:10.1155/2020/2503790)

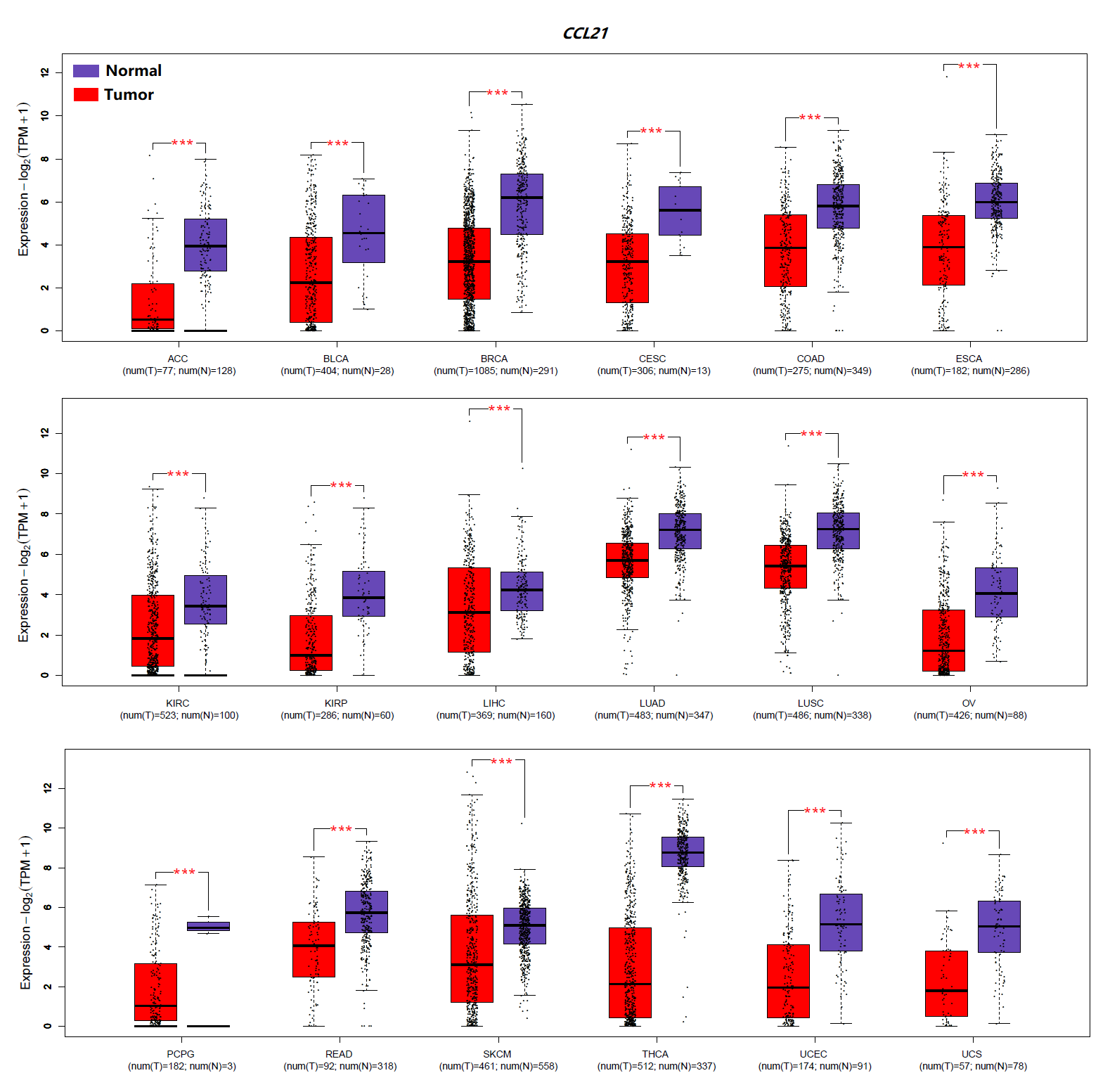

Supplement: Supplementary Materials — Table S1. Sample size of cancer and normal tissues in the datasets used in this study. Figure S1. CCL21 tends to be downregulated in cancer. Figure S2. CBFA2T3 tends to be downregulated in cancer. Figure S3. XPNPEP2 tends to be downregulated in cancer. Figure S4. Downregulation of tumor suppressor genes (TSGs) is associated with a worse survival prognosis in various cancers. Figure S5. The methylation levels of RASGRP2 promoter are higher in various cancer types than in normal tissues. Figure S6. The methylation levels of RASGRP2 promoter are inversely associated with the expression levels of RASGRP2 in cancer. Figure S7. The CCL21 promoter methylation levels are significantly upregulated in 16 TCGA cancer types compared to their normal tissues. Figure S8. Correlations of the expression levels of CCL21 with tumor purity in cancers. Figure S9. Correlations of the expression levels of CCL21 with immune cell infiltration levels in cancers. Figure S10. Correlation of tumor purity with survival prognosis in cancer. [file 2503790.f1.zip › 2503790.f1/Figure S1.tif]

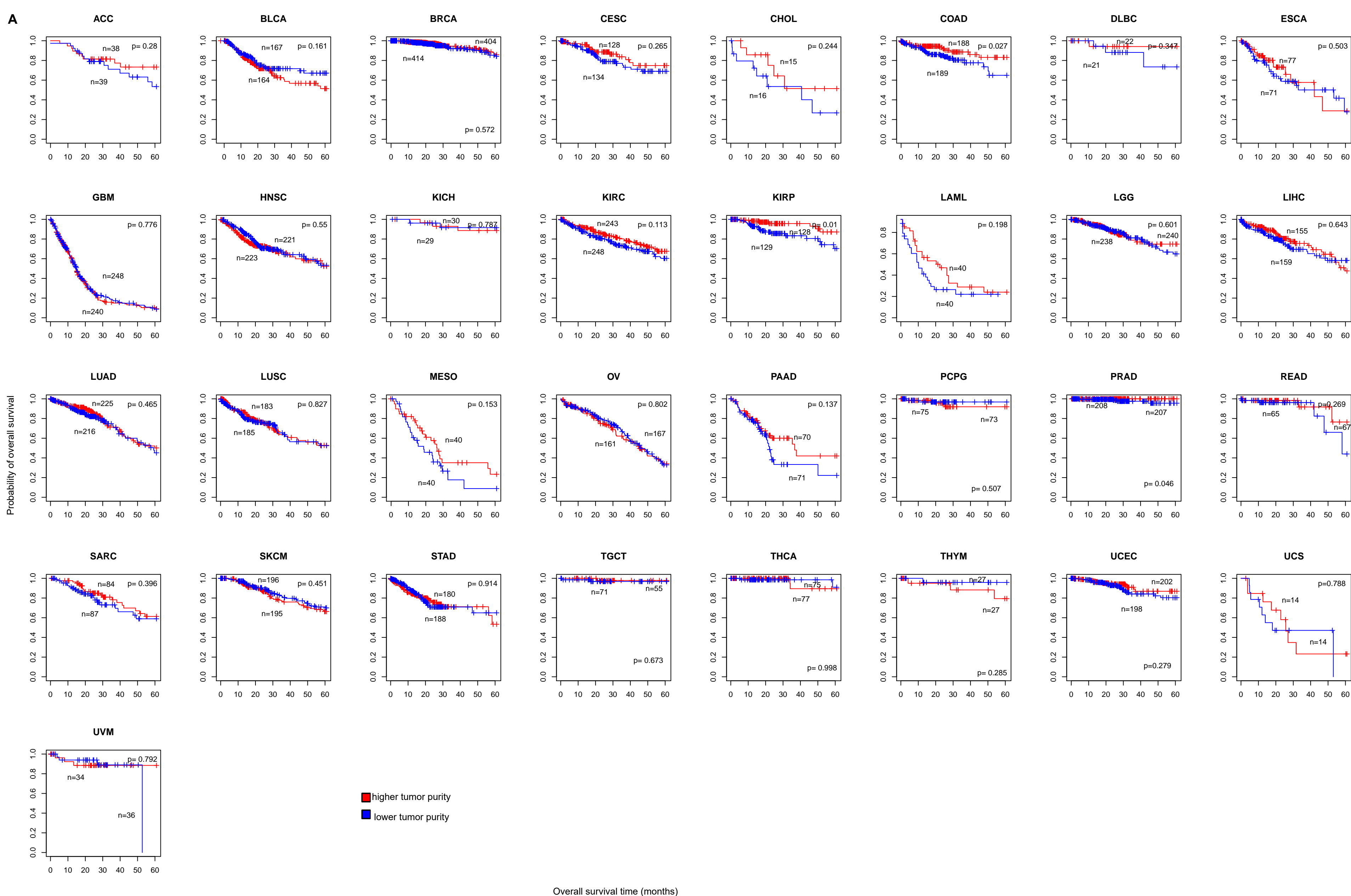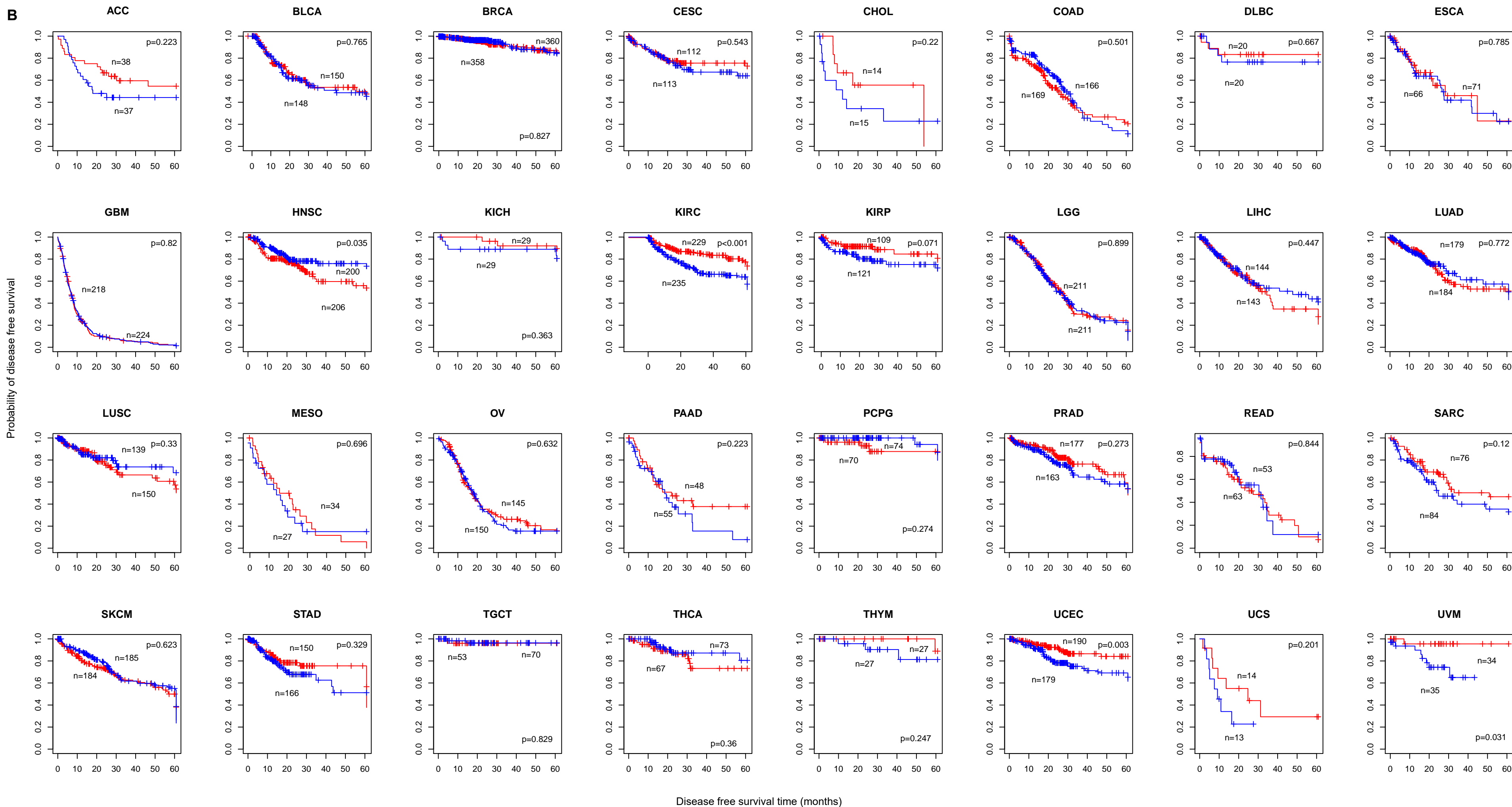

Supplement: Supplementary Materials — Table S1. Sample size of cancer and normal tissues in the datasets used in this study. Figure S1. CCL21 tends to be downregulated in cancer. Figure S2. CBFA2T3 tends to be downregulated in cancer. Figure S3. XPNPEP2 tends to be downregulated in cancer. Figure S4. Downregulation of tumor suppressor genes (TSGs) is associated with a worse survival prognosis in various cancers. Figure S5. The methylation levels of RASGRP2 promoter are higher in various cancer types than in normal tissues. Figure S6. The methylation levels of RASGRP2 promoter are inversely associated with the expression levels of RASGRP2 in cancer. Figure S7. The CCL21 promoter methylation levels are significantly upregulated in 16 TCGA cancer types compared to their normal tissues. Figure S8. Correlations of the expression levels of CCL21 with tumor purity in cancers. Figure S9. Correlations of the expression levels of CCL21 with immune cell infiltration levels in cancers. Figure S10. Correlation of tumor purity with survival prognosis in cancer. [file 2503790.f1.zip › 2503790.f1/Figure S10.pdf]

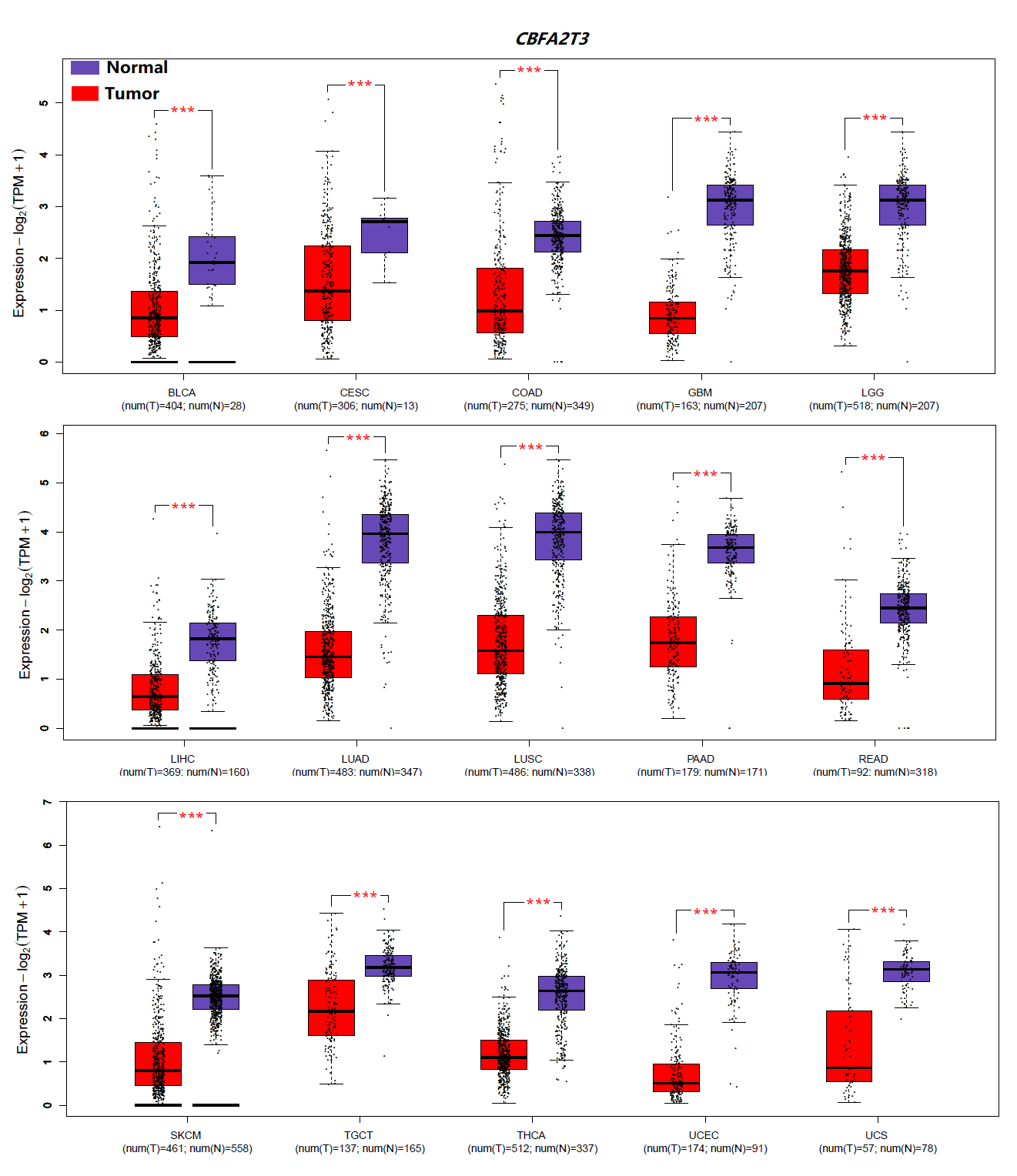

Supplement: Supplementary Materials — Table S1. Sample size of cancer and normal tissues in the datasets used in this study. Figure S1. CCL21 tends to be downregulated in cancer. Figure S2. CBFA2T3 tends to be downregulated in cancer. Figure S3. XPNPEP2 tends to be downregulated in cancer. Figure S4. Downregulation of tumor suppressor genes (TSGs) is associated with a worse survival prognosis in various cancers. Figure S5. The methylation levels of RASGRP2 promoter are higher in various cancer types than in normal tissues. Figure S6. The methylation levels of RASGRP2 promoter are inversely associated with the expression levels of RASGRP2 in cancer. Figure S7. The CCL21 promoter methylation levels are significantly upregulated in 16 TCGA cancer types compared to their normal tissues. Figure S8. Correlations of the expression levels of CCL21 with tumor purity in cancers. Figure S9. Correlations of the expression levels of CCL21 with immune cell infiltration levels in cancers. Figure S10. Correlation of tumor purity with survival prognosis in cancer. [file 2503790.f1.zip › 2503790.f1/Figure S2.tif]

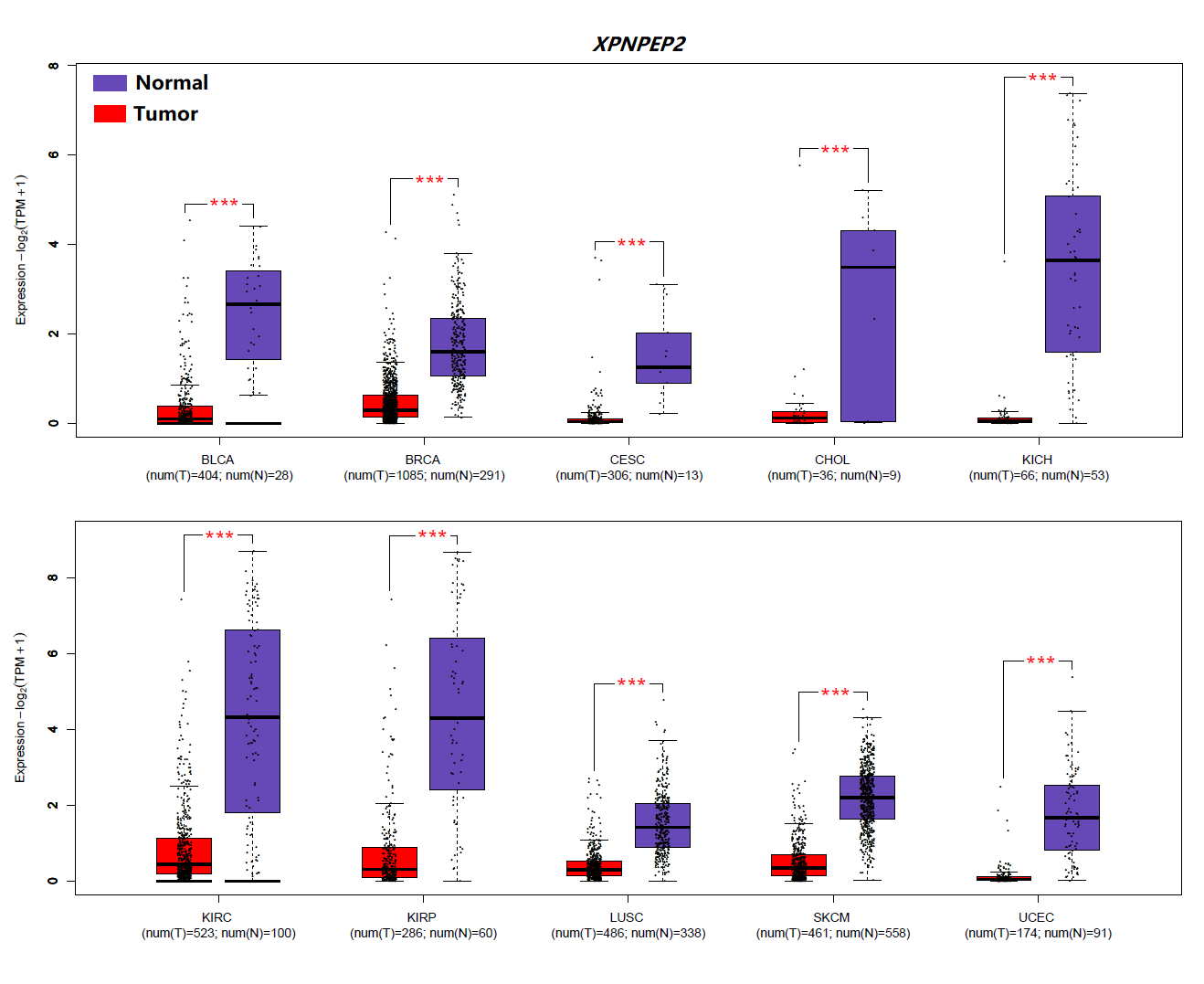

Supplement: Supplementary Materials — Table S1. Sample size of cancer and normal tissues in the datasets used in this study. Figure S1. CCL21 tends to be downregulated in cancer. Figure S2. CBFA2T3 tends to be downregulated in cancer. Figure S3. XPNPEP2 tends to be downregulated in cancer. Figure S4. Downregulation of tumor suppressor genes (TSGs) is associated with a worse survival prognosis in various cancers. Figure S5. The methylation levels of RASGRP2 promoter are higher in various cancer types than in normal tissues. Figure S6. The methylation levels of RASGRP2 promoter are inversely associated with the expression levels of RASGRP2 in cancer. Figure S7. The CCL21 promoter methylation levels are significantly upregulated in 16 TCGA cancer types compared to their normal tissues. Figure S8. Correlations of the expression levels of CCL21 with tumor purity in cancers. Figure S9. Correlations of the expression levels of CCL21 with immune cell infiltration levels in cancers. Figure S10. Correlation of tumor purity with survival prognosis in cancer. [file 2503790.f1.zip › 2503790.f1/Figure S3.tif]

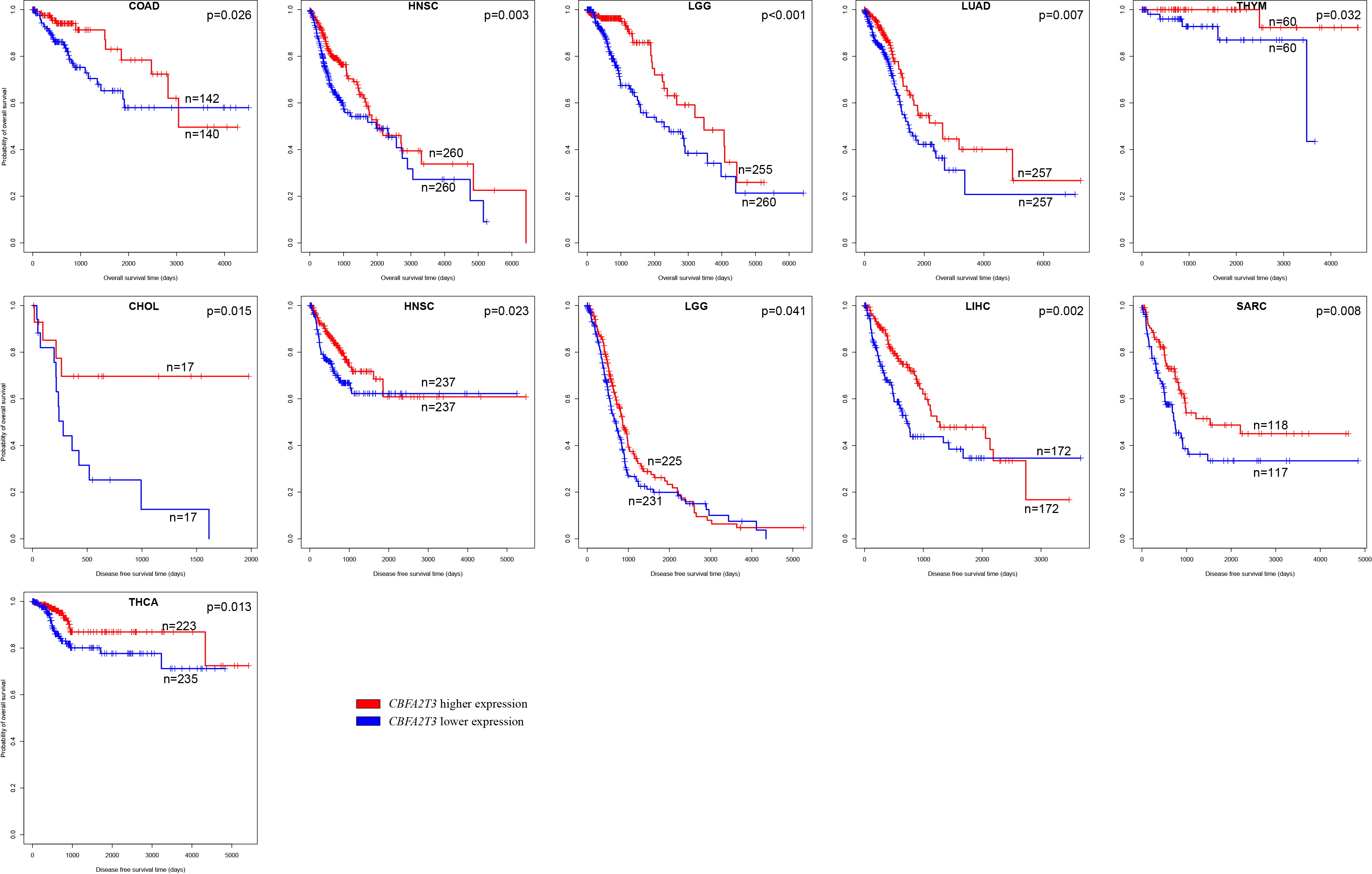

Supplement: Supplementary Materials — Table S1. Sample size of cancer and normal tissues in the datasets used in this study. Figure S1. CCL21 tends to be downregulated in cancer. Figure S2. CBFA2T3 tends to be downregulated in cancer. Figure S3. XPNPEP2 tends to be downregulated in cancer. Figure S4. Downregulation of tumor suppressor genes (TSGs) is associated with a worse survival prognosis in various cancers. Figure S5. The methylation levels of RASGRP2 promoter are higher in various cancer types than in normal tissues. Figure S6. The methylation levels of RASGRP2 promoter are inversely associated with the expression levels of RASGRP2 in cancer. Figure S7. The CCL21 promoter methylation levels are significantly upregulated in 16 TCGA cancer types compared to their normal tissues. Figure S8. Correlations of the expression levels of CCL21 with tumor purity in cancers. Figure S9. Correlations of the expression levels of CCL21 with immune cell infiltration levels in cancers. Figure S10. Correlation of tumor purity with survival prognosis in cancer. [file 2503790.f1.zip › 2503790.f1/Figure S4.tif]

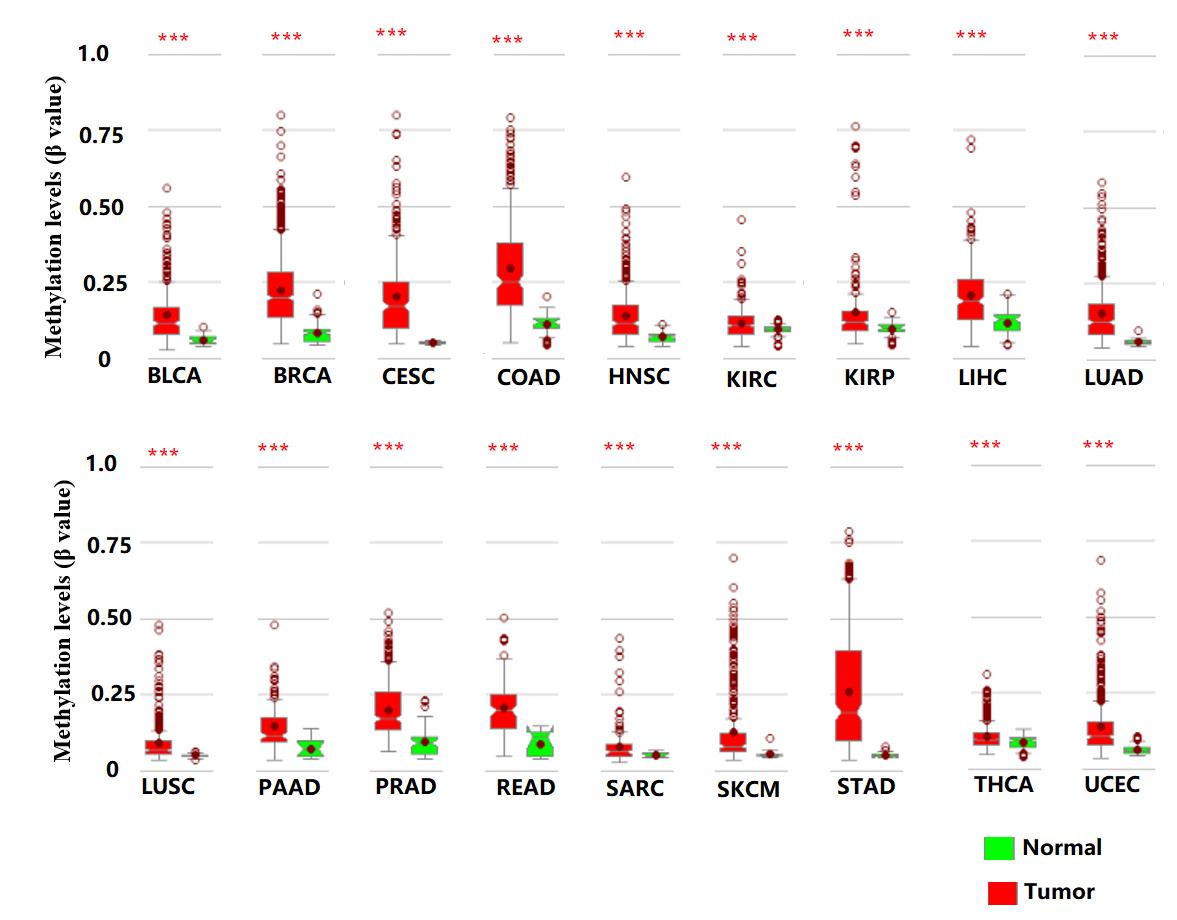

Supplement: Supplementary Materials — Table S1. Sample size of cancer and normal tissues in the datasets used in this study. Figure S1. CCL21 tends to be downregulated in cancer. Figure S2. CBFA2T3 tends to be downregulated in cancer. Figure S3. XPNPEP2 tends to be downregulated in cancer. Figure S4. Downregulation of tumor suppressor genes (TSGs) is associated with a worse survival prognosis in various cancers. Figure S5. The methylation levels of RASGRP2 promoter are higher in various cancer types than in normal tissues. Figure S6. The methylation levels of RASGRP2 promoter are inversely associated with the expression levels of RASGRP2 in cancer. Figure S7. The CCL21 promoter methylation levels are significantly upregulated in 16 TCGA cancer types compared to their normal tissues. Figure S8. Correlations of the expression levels of CCL21 with tumor purity in cancers. Figure S9. Correlations of the expression levels of CCL21 with immune cell infiltration levels in cancers. Figure S10. Correlation of tumor purity with survival prognosis in cancer. [file 2503790.f1.zip › 2503790.f1/Figure S5.tif]

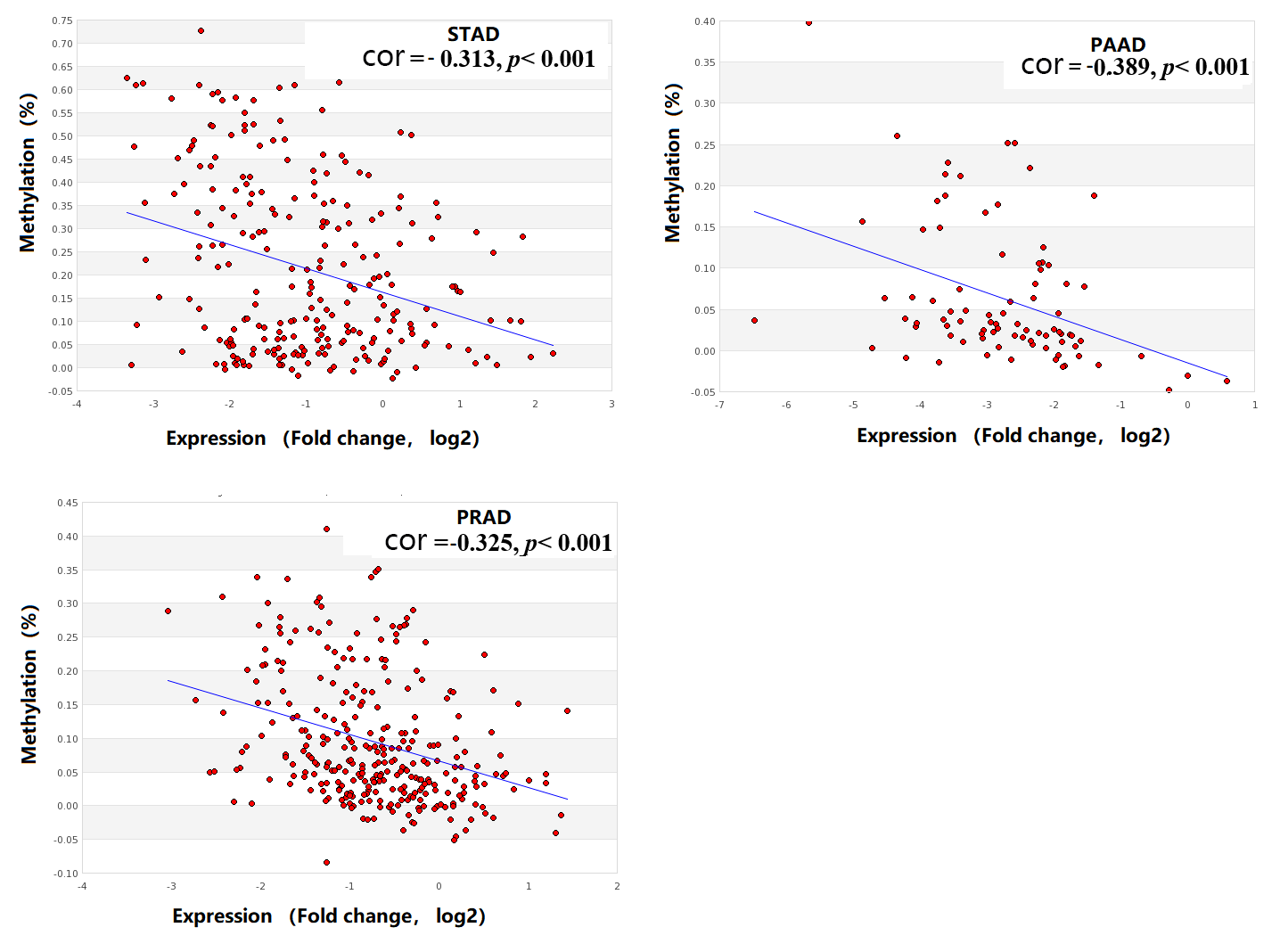

Supplement: Supplementary Materials — Table S1. Sample size of cancer and normal tissues in the datasets used in this study. Figure S1. CCL21 tends to be downregulated in cancer. Figure S2. CBFA2T3 tends to be downregulated in cancer. Figure S3. XPNPEP2 tends to be downregulated in cancer. Figure S4. Downregulation of tumor suppressor genes (TSGs) is associated with a worse survival prognosis in various cancers. Figure S5. The methylation levels of RASGRP2 promoter are higher in various cancer types than in normal tissues. Figure S6. The methylation levels of RASGRP2 promoter are inversely associated with the expression levels of RASGRP2 in cancer. Figure S7. The CCL21 promoter methylation levels are significantly upregulated in 16 TCGA cancer types compared to their normal tissues. Figure S8. Correlations of the expression levels of CCL21 with tumor purity in cancers. Figure S9. Correlations of the expression levels of CCL21 with immune cell infiltration levels in cancers. Figure S10. Correlation of tumor purity with survival prognosis in cancer. [file 2503790.f1.zip › 2503790.f1/Figure S6.tif]

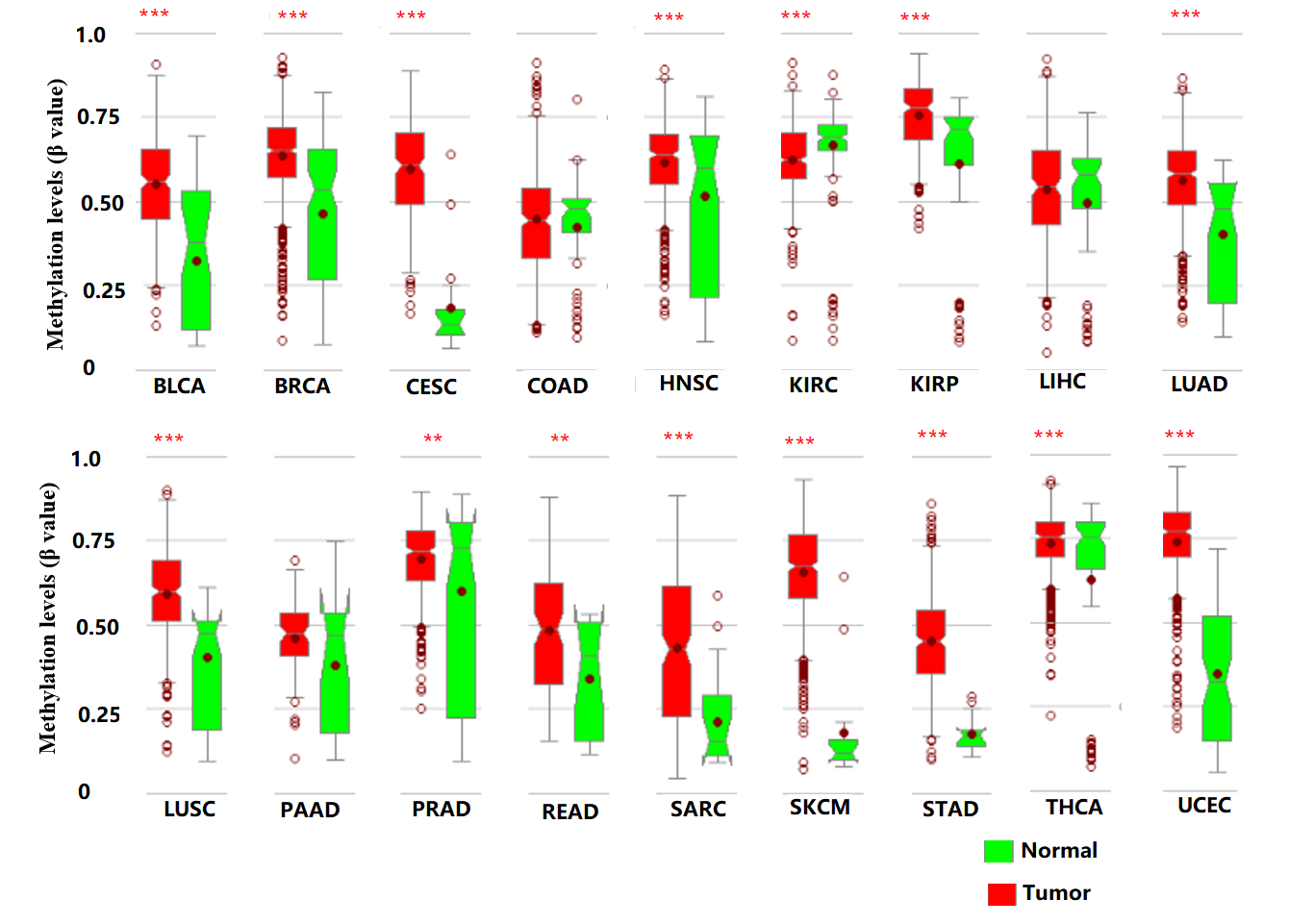

Supplement: Supplementary Materials — Table S1. Sample size of cancer and normal tissues in the datasets used in this study. Figure S1. CCL21 tends to be downregulated in cancer. Figure S2. CBFA2T3 tends to be downregulated in cancer. Figure S3. XPNPEP2 tends to be downregulated in cancer. Figure S4. Downregulation of tumor suppressor genes (TSGs) is associated with a worse survival prognosis in various cancers. Figure S5. The methylation levels of RASGRP2 promoter are higher in various cancer types than in normal tissues. Figure S6. The methylation levels of RASGRP2 promoter are inversely associated with the expression levels of RASGRP2 in cancer. Figure S7. The CCL21 promoter methylation levels are significantly upregulated in 16 TCGA cancer types compared to their normal tissues. Figure S8. Correlations of the expression levels of CCL21 with tumor purity in cancers. Figure S9. Correlations of the expression levels of CCL21 with immune cell infiltration levels in cancers. Figure S10. Correlation of tumor purity with survival prognosis in cancer. [file 2503790.f1.zip › 2503790.f1/Figure S7.tif]

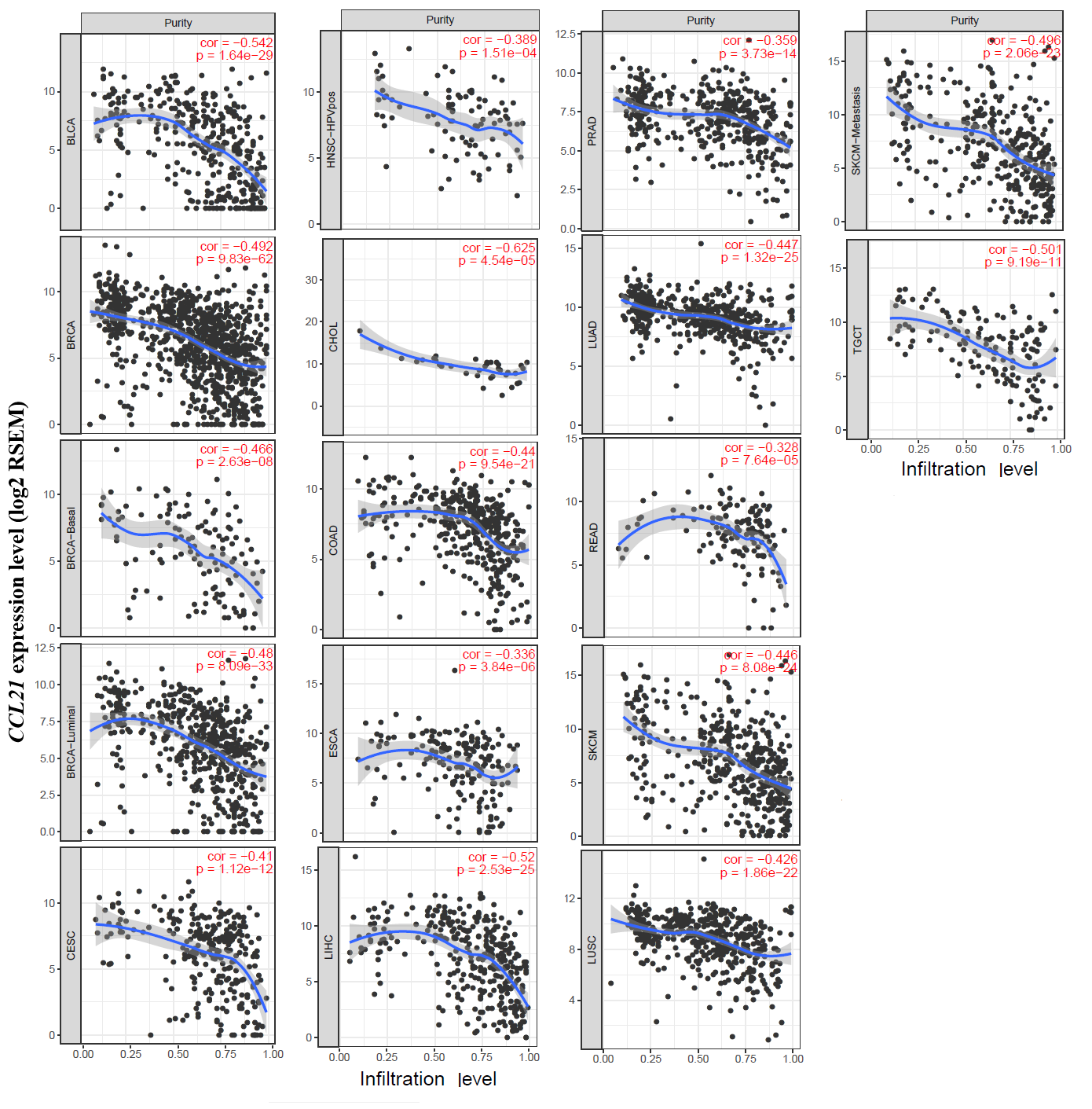

Supplement: Supplementary Materials — Table S1. Sample size of cancer and normal tissues in the datasets used in this study. Figure S1. CCL21 tends to be downregulated in cancer. Figure S2. CBFA2T3 tends to be downregulated in cancer. Figure S3. XPNPEP2 tends to be downregulated in cancer. Figure S4. Downregulation of tumor suppressor genes (TSGs) is associated with a worse survival prognosis in various cancers. Figure S5. The methylation levels of RASGRP2 promoter are higher in various cancer types than in normal tissues. Figure S6. The methylation levels of RASGRP2 promoter are inversely associated with the expression levels of RASGRP2 in cancer. Figure S7. The CCL21 promoter methylation levels are significantly upregulated in 16 TCGA cancer types compared to their normal tissues. Figure S8. Correlations of the expression levels of CCL21 with tumor purity in cancers. Figure S9. Correlations of the expression levels of CCL21 with immune cell infiltration levels in cancers. Figure S10. Correlation of tumor purity with survival prognosis in cancer. [file 2503790.f1.zip › 2503790.f1/Figure S8.tif]

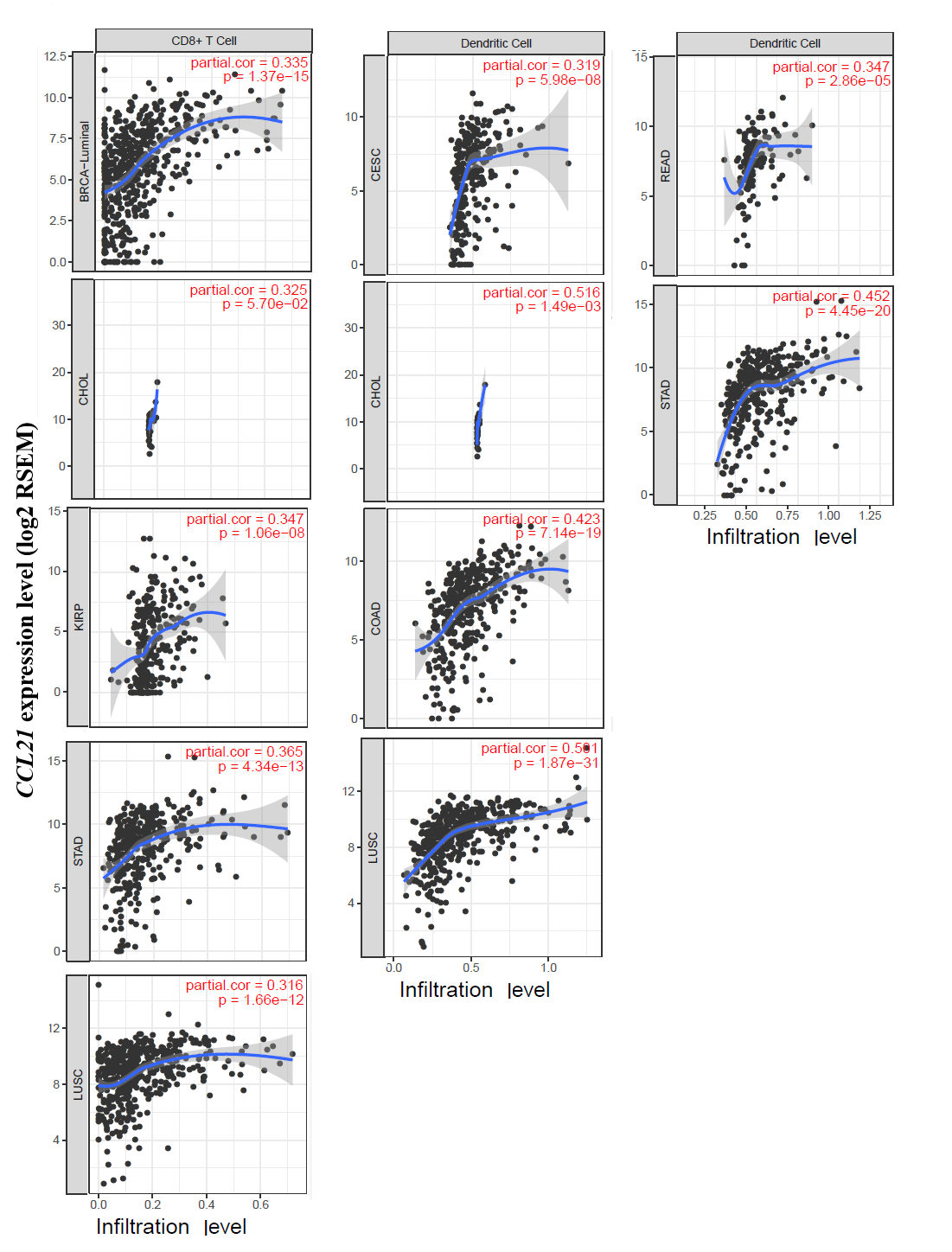

Supplement: Supplementary Materials — Table S1. Sample size of cancer and normal tissues in the datasets used in this study. Figure S1. CCL21 tends to be downregulated in cancer. Figure S2. CBFA2T3 tends to be downregulated in cancer. Figure S3. XPNPEP2 tends to be downregulated in cancer. Figure S4. Downregulation of tumor suppressor genes (TSGs) is associated with a worse survival prognosis in various cancers. Figure S5. The methylation levels of RASGRP2 promoter are higher in various cancer types than in normal tissues. Figure S6. The methylation levels of RASGRP2 promoter are inversely associated with the expression levels of RASGRP2 in cancer. Figure S7. The CCL21 promoter methylation levels are significantly upregulated in 16 TCGA cancer types compared to their normal tissues. Figure S8. Correlations of the expression levels of CCL21 with tumor purity in cancers. Figure S9. Correlations of the expression levels of CCL21 with immune cell infiltration levels in cancers. Figure S10. Correlation of tumor purity with survival prognosis in cancer. [file 2503790.f1.zip › 2503790.f1/Figure S9.tif]
